# Detection of SARS-CoV-2 RNA and Antibodies in Diverse Samples: Protocol to Validate the Sufficiency of Provider-Observed, Home-Collected Blood, Saliva, and Oropharyngeal Samples

**DOI:** 10.2196/19054

**Published:** 2020-04-24

**Authors:** Patrick Sean Sullivan, Charles Sailey, Jodie Lynn Guest, Jeannette Guarner, Colleen Kelley, Aaron Julius Siegler, Mariah Valentine-Graves, Laura Gravens, Carlos del Rio, Travis Howard Sanchez

**Affiliations:** 1 Department of Epidemiology Rollins School of Public Health Emory University Atlanta, GA United States; 2 Molecular Testing Labs Vancouver, WA United States; 3 Division of Infectious Diseases Emory University School of Medicine Atlanta, GA United States; 4 Department of Behavioral Sciences and Health Education Rollins School of Public Health Emory University Atlanta, GA United States

**Keywords:** SARS-CoV-2, RNA-PCR, serology, COVID-19, PCR, public health, outbreak, infectious disease, diagnostic, telemedicine, testing

## Abstract

**Background:**

The response in the United States to the coronavirus disease (COVID-19) pandemic has been hampered by a lack of aggressive testing for the infection. Testing for severe acute respiratory syndrome coronavirus 2 (SARS-CoV-2) is the cornerstone of an effective public health response. However, efforts to test have been hampered by limited reagents, limitations in the availability of swabs used for the collection of nasopharyngeal swab (NPS) specimens, limitations in personal protective equipment (PPE) for health care providers collecting the NPS specimens, and limitations in viral transport media for transporting the specimens. Therefore, more flexible options for screening for SARS-CoV-2 RNA and serologic responses are critical to inform clinical and public health responses.

**Objective:**

We aim to document the ability of patients to self-collect sufficient specimens for SARS-CoV-2 viral detection and serology.

**Methods:**

Patient self-collection of samples will be done with observation by a health care provider during a telemedicine session. Participants will be mailed a specimen collection kit, engage in a telehealth session with a provider through a HIPPA (Health Insurance Portability and Accountability Act of 1996)-compliant video meeting, and collect specimens while being observed by the provider. Providers will record whether they are confident in the suitability of the specimen for laboratory testing that would inform clinical decision making. We will objectively assess the sufficiency of biological material in the mailed-in specimens.

**Results:**

The protocol was approved by the Emory University Institutional Review Board (IRB) on March 30, 2020 (Protocol number 371). To date, we have enrolled 159 participants.

**Conclusions:**

Defining a conceptual framework for assessing the sufficiency of patient-collected samples for the detection of SARS-CoV-2 RNA and serologic responses to infection is critical for facilitating public health responses and providing PPE-sparing options to increase testing. Validation of alternative methods of specimen collection should include objective measures of the sufficiency of specimens for testing. A strong evidence base for diversifying testing modalities will improve tools to guide public health responses to the COVID-19 pandemic.

## Introduction

### Background

The global pandemic of SARS-CoV-2 (severe acute respiratory syndrome coronavirus 2) infection and associated illness (coronavirus disease or COVID-19) have emerged very quickly, challenging traditional systems of clinical and public health response [1,2]. There is broad consensus that adequate testing for SARS-CoV-2 is imperative as a cornerstone of public health efforts to control the spread of the virus [3-5]. The response in the United States to the SARS-CoV-2 pandemic has been hampered by a slow implementation of screening programs and by a variety of factors that have limited the extent of SARS-CoV-2 testing. Following initial problems with quality control of reagents [6], the US government allowed more liberal policies for the development of laboratory developed tests (LDTs) [7], which allowed for a massive expansion of capacity in terms of availability of equipment and staff capacity at commercial laboratories.

However, other factors now limit the reach and volume of testing for SARS-CoV-2 infection. Currently, the gold standard specimen for testing for SARS-CoV-2 RNA is a provider-administered nasopharyngeal swab (NPS) [8]; there are currently supply chain challenges, including shortages of rigid swabs for NPS collection, personal protective equipment (PPE) required for health care workers to collect NPS specimens, and viral transport media required for transporting specimens [9-12]. There are also important questions about testing sites for SARS-CoV-2 infection—should people with mild symptoms who may or may not have COVID-19 come into clinics for testing if they do not require immediate clinical care? How can people who have been quarantined receive testing to document viral shedding to guide the time of release from quarantine, without coming into places where patients are congregating? Finally, we have essentially no epidemiologic data about asymptomatic infection, which could be answered with serology data. Decisions about when to end “stay at home” curfews should likely be based, in part, on the prevalence of antibodies (and, perhaps, immunity) among populations. All these applications will require mechanisms to collect specimens that minimize the need for PPE and allow flexibility of where specimens are collected. Patient-collected samples have appeal in terms of minimizing PPE requirements and enabling the possibility of epidemiologic studies to characterize the infection and immune status of populations.

The regulatory environment governing both the development of LDTs and specifically the testing of patient-collected samples, either in the office of the health care provider or at home, is complicated [13,14]. In response to the urgency of COVID-19 in the United States, the Food and Drug Administration (FDA) has relaxed the regulatory pathways for the development of LDTs to increase the national laboratory capacity [15]. With respect to specimen types, the FDA has developed two pathways for approval for SARS-CoV-2 infection testing: (1) develop laboratory data documenting validation or performance to submit to the FDA for review as an Emergency Use Authorization (EUA); or (2) use state regulatory mechanisms when the test is developed under the jurisdiction of the state in which the lab resides. In the latter case, the state regulators take responsibility for COVID-19 testing by laboratories in their state.

Patient-collected samples have slightly different requirements. The FDA has issued clarifications that the ability of states to oversee the validation of LDTs with patient-collected samples is not covered by the general policy for the development of LDTs, and that assays with patient-collected samples will be required to submit data for review through the FDA for an EUA application [15]. The FDA recently updated guidance to allow collection of patient-collected mid-nasal turbinate samples collected in the provider office, but specifically noted that this approval did not extend to patient-collected samples collected at home [16]. Guidelines for the validation of patient-collected serology specimens do not appear to be explicitly addressed under current FDA guidance, which has focused on the collection of samples for viral detection.

Outside of the realm of SARS-CoV-2 diagnosis, the FDA has reviewed and approved patient-collected samples for a wide variety of laboratory assays, including HIV serology through dried blood spot (DBS) specimens [17]. In other fields, there is a long history of using patient-collected samples under research protocols to develop data on acceptability and to provide clinical services (eg, STI [sexually transmitted infection] testing) as part of research studies [18,19].

We anticipate that there will be biological, immunologic, and temporal aspects that will be important to consider in the design of validation studies for alternative specimen types and results interpretation. When directly comparing provider-collected and patient-collected samples of the same type (eg, provider-collected versus patient-collected oropharyngeal swab [OPS]), it is appropriate to compare the cycle threshold (C_t_) for the paired samples to assess comparability. However, RNA concentrations may differ between different specimen types because of differences in RNA shedding at the two sites, such that a direct comparison of C_t_ results between nasopharyngeal and oropharyngeal swabs might not be an appropriate comparison. Similarly, the timing of onset and waning of IgM (immunoglobulin M) titers in patients and detection of IgA (immunoglobulin A) in saliva or serum following infection with SARS-CoV-2 are not well understood. Finally, it is unclear whether RNA might persist for variable lengths of time after infection in different specimen types. For example, To et al [20] document the presence of RNA in saliva for nearly 2 weeks post hospitalization. Ultimately, all of these questions need to be explored to make the most evidence-based recommendations for specimen screening types and collection methods in specific time phases of the infection cycle.

### Objective

In this paper, we lay out a protocol for describing the sufficiency of patient-collected samples for SARS-CoV-2 infection testing in OPS and saliva, and for immune response to SARS-CoV-2 in DBS and saliva. We consider two aspects of assessment:

1. Do providers who observe patient-collected samples consider them to be comparable to provider-collected specimens in terms of *specimen suitability* for testing for SARS-CoV-2 RNA and antibodies?
2. Do assessments of specimen quality (eg, human nucleic acid for OPS and saliva, specimen saturation and DBS size for DBS cards) document that patient-collected samples contain *sufficient biological material* for accurate testing?

## Methods

We propose methods to validate multiple sample types for RNA-PCR (polymerase chain reaction) and for serology tests. Proposed specimen types and assays are depicted in Table 1.

**Table 1:** Specimen types and assays to be performed in an evaluation of diverse samples for SARS-CoV-2 (severe acute respiratory syndrome coronavirus 2) RNA and antibody testing.

Specimen	Oropharyngeal swab	Saliva	Dried blood spot
SARS-CoV-2 RNA	✔	✔	
IgG^a^		✔	✔
IgM^b^		✔	✔
IgA^c^		✔	✔

^a^IgG: immunoglobulin G.

^b^IgM: immunoglobulin M.

^c^IgA: immunoglobulin A.

### Specimen Collection

#### Oropharyngeal Swab Self-Collection

**Figure 1:**
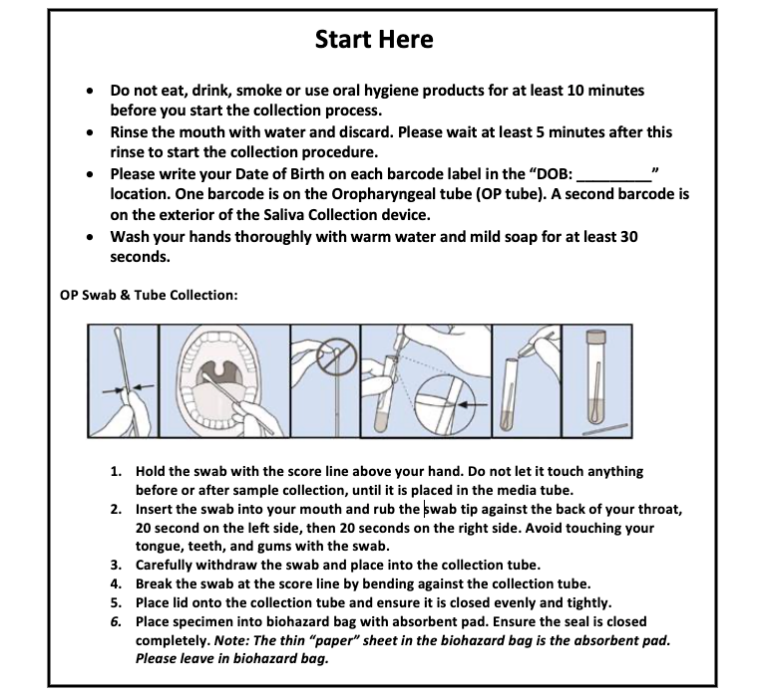
Participant instructions for self-collection via oropharyngeal swab.

Patients will be provided with printed instructions for collection (Figure 1). They will be instructed to insert the swab into their mouth and rub the swab tip against the back of their throat for 20 seconds on the left side, then 20 seconds on the right side. They are advised to avoid touching their tongue, teeth, and gums with the swab. They are instructed to insert the swab in the tube of viral transport media, break the swab at the score, and cap the tube.

#### Self-Collection of Dried Blood Spots

**Figure 2:**
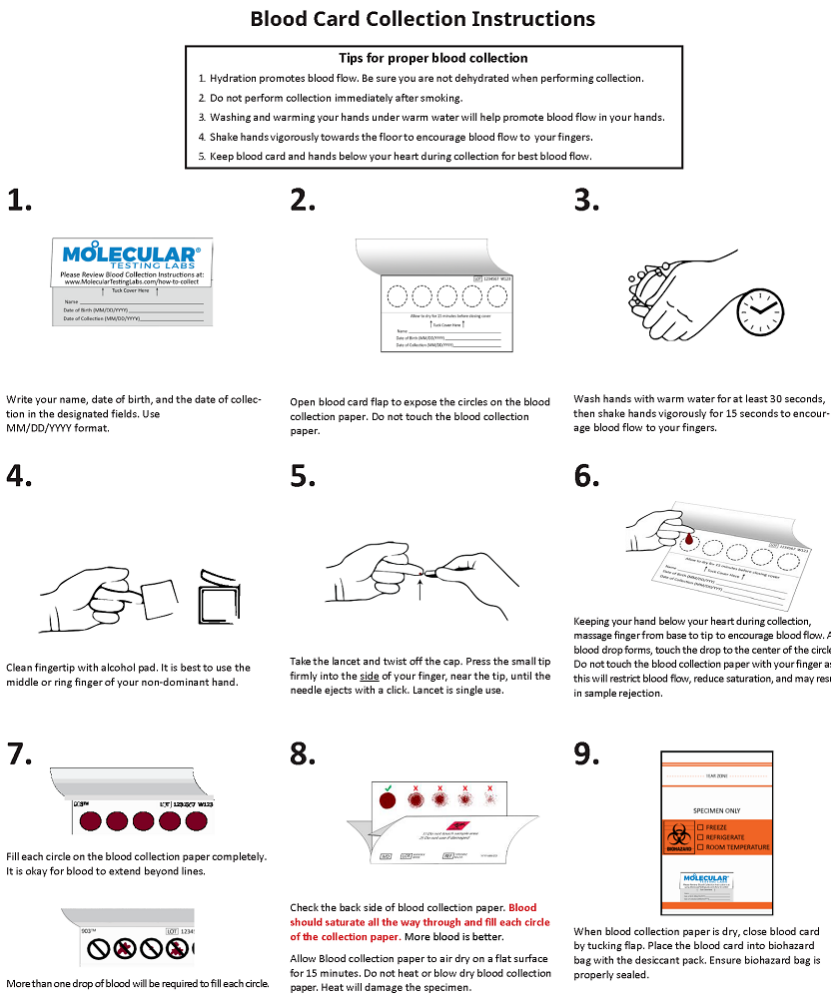
Participant instructions for self-collection of dried blood spots.

Patients will be provided with printed instructions for DBS collection (Figure 2). They will be instructed to wash their hands thoroughly, use an alcohol swab to clean the tip of the middle or ring finger of their nondominant hand, release the blade of the provided lancet by pressing into the side of the finger near the tip of the finger, and fill each of the 5 circles on a Whatman standard dried blood spot collection card. Participants are instructed to allow the blood card to dry for at least 15 minutes before packaging for return shipment.

#### Saliva Self-Collection

**Figure 3:**
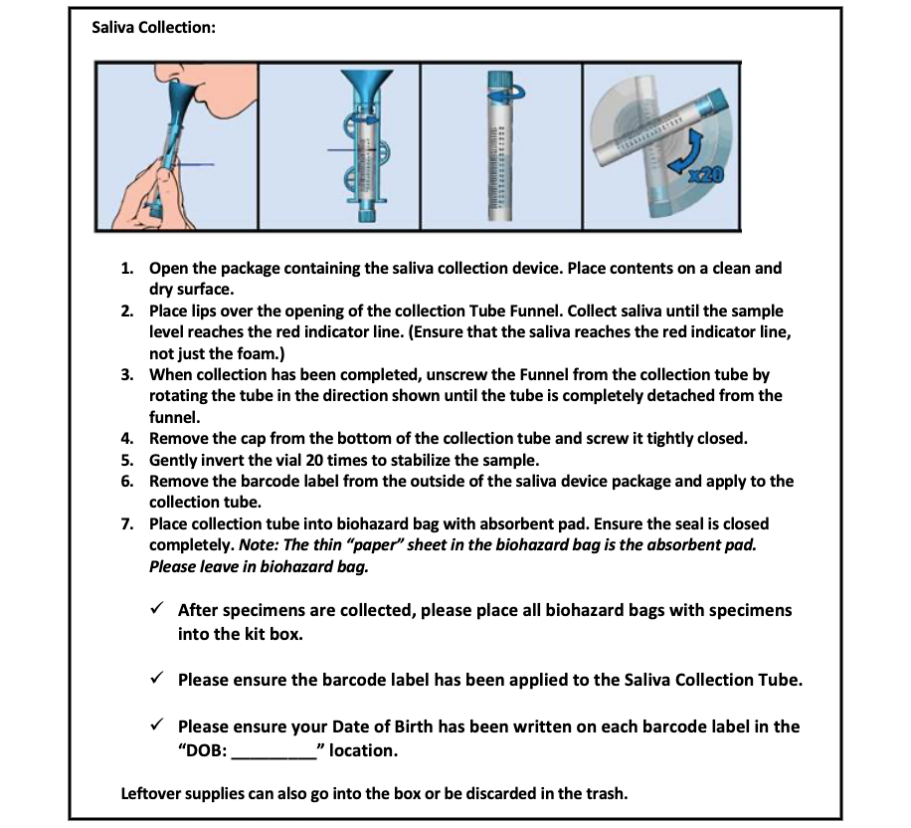
Participant instructions for self-collection of saliva.

Participants are asked not to eat, drink, smoke or use oral hygiene products for at least 10 minutes before the collection process (Figure 3). Participants will be instructed to rinse their mouth with water and discard, and then wait 5 minutes after the rinse before collecting the specimen. Participants will be instructed to place their lips over the collection tube funnel and collect saliva until the saliva reaches the red indicator line. They will then be instructed to screw the cap on the tube tightly and invert it 20 times to stabilize the sample. The participant will be instructed to write their date of birth on the tube before preparing for shipping.

### Provider Observations

Providers will observe participants as they collect specimens through a telehealth video session on a HIPPA (Health Insurance Portability and Accountability Act of 1996)-compliant video conference service. After establishing a secure connection and confirming the identity of participants using a study identifier, the provider will direct the participant to use the provided instructions to collect the specimen; the provider will identify their role as that of an observer. The provider will document on case report forms (CRFs) that they observed the participant collecting the specimens (see Multimedia Appendix 1 for CRFs). The provider will also record their assessment of whether the specimen collected is suitable for laboratory testing, and, as secondary assessments, complete checklists of steps in the correct collection of the specimen (eg, did not smoke or drink while collecting saliva specimen, inverted closed saliva tube 20 times as directed, dropped blood on the DBS card rather than touching the card).

### Testing

#### RNA-PCR

Specimens will first be checked for quality. The samples will then undergo total nucleic acid extraction using the Thermo Kingfisher platform (Fisher Scientific). Isolated RNA will be reverse transcribed to DNA using a one-step, one-tube system via reagents from Thermo (Fisher Scientific). The second half of the one-tube system will involve qPCR (quantitative polymerase chain reaction). The reverse-transcribed DNA will undergo qPCR with primers and probes targeting 3 gene regions of the SARS-CoV-2 genome (N, S, ORF1), using reagents from Thermo. The results will be analyzed, and an interpretation will be made based on C_t_ values and positive identification of the nucleic acid.

#### Specimen Sufficiency for RNA-PCR

We will test OPS and saliva specimens for RNase P (ribonuclease P) as an endogenous internal amplification control and to quantify the nucleic acid content of the specimen [21]. We will consider OPS and saliva specimens with C_t_ values of <30 to indicate sufficient collection of biological material in the saliva sample and the swab.

#### Serology Tests

Specimens will first be checked for quality. For blood, a 6 mm punch will be obtained from the DBS, and the material will undergo standard antibody extraction using Tris buffer. For saliva, the sample will be aliquoted and used directly in the serology assay. Once the material is added to the reaction tube, the enzyme immunoassay primary and secondary antibodies (SARS-CoV-2 assay, IgG and IgM, Epitope Diagnostics; IgA, EuroIMMUN) will be added using an automated liquid handler instrument (DSX; Dynex Technologies). The protocol will follow the manufacturer’s guidelines for reaction conditions, data interpretation, and ensuring that internal controls pass.

#### Specimen Sufficiency for Serology

For DBSs, we will conduct a three-point quality check, documenting the visual appearance of the blood spot, whether it is soaked through the paper, and whether the circles are filled, as we have previously reported [22].

### Analysis

We will tabulate the provider impressions of specimen suitability (primary outcome); exploratory analyses for provider observation will include enumeration of how common certain errors in self-collection were. For assessment of the sufficiency of RNA-PCR specimens, we will tabulate the proportion of OPS and saliva samples that had C_t_ values for RNAse P<30. As a secondary analysis, we will examine whether the RNAse P C_t_ values for the patient-collected OPS were different from the C_t_ values from a historical set of provider-collected OPS tested with the same reagents and laboratory equipment in the study laboratory. These analyses will involve comparing the means of the two groups using a *t* test.

## Results

The protocol was approved by the Emory University Institutional Review Board (IRB) on March 30, 2020 (Protocol number 371). To date, we have enrolled 159 participants.

## Discussion

There is an urgent need to develop and validate new methods to monitor SARS-CoV-2 infection status and immune experience [4]. Currently, provider and supply chain shortages threaten our national capacity to diagnose people who need care and monitor the growing COVID-19 pandemic. Patient-collected samples, if they are validated and approved through regulatory channels for clinical purposes, offer several advantages from clinical and public health perspectives. From a clinical perspective, patient-collected specimen options will decrease provider burden, allow for follow-up monitoring for viral shedding without the need for return office visits, and reduce risks for provider exposure during specimen collection. From a supply chain perspective, depending on the specimen that is used, self-collection can reduce the need for PPE for providers who would otherwise collect the sample, will reduce the need for rigid NPSs, and could reduce the need for viral transport media (eg, saliva samples). From a public health perspective, having options for patient-collected samples will allow for population-based studies to measure the population prevalence of current and past infection with SARS-CoV-2. Such studies are critical to understand the natural history of infection, to develop an understanding of what proportion of the population have asymptomatic infections, to monitor population immunity, and to reach patients who live in remote areas with testing.

We developed this protocol for validation, recognizing the extreme urgency of developing new testing options and appreciating the regulatory structures that ensure that clinical testing in the United States meets high standards and produces actionable results. We believe that having providers observe patients collecting specimens is an important steppingstone on the path between relying wholly on provider-collected samples (and the required PPE and clinical visits) and the use of patient-collected samples collected outside of the supervision of providers. We note that the FDA has approved SARS-CoV-2 testing on patient-collected mid-nasal turbinate swabs, but only if the patient-collected swabs are collected in the provider’s office [16]. The kappa values of the mid-nasal turbinate study have not been reported, but the sensitivity of the patient-collected swabs to detect SARS-CoV-2 RNA among those known to be infected was 90% [16]. This approval is a rational decision, because modeling data suggest that testing at this stage of the epidemic is still valuable in blunting it, even if it is imperfect [3]. Recent data suggest that staff-collected and patient-collected mid-nasal turbinate swabs have high correlation for the detection of influenza viruses [23].

The COVID-19 pandemic has been remarkable for its rapid onset and spread into new populations. The public health and clinical medicine systems in the United States have not had time to respond in conventional ways to this pandemic. There is a need to be innovative in developing and deploying new strategies to meet the clinical needs of patients who are infected with SARS-CoV-2 and simultaneously to gather data to understand the broad picture of the epidemic and to monitor infections and immunity at the population level. Given the catastrophic demands on our hospitals and medical offices, we must develop ways to move testing for screening purposes and epidemiologic monitoring out of the health care system [24]. Patient-collected specimens are widely used for monitoring of other infectious diseases and health conditions, and it is imperative to validate and deploy self-collection tools to understand and respond to this pandemic. We propose a structured and objective process by which patient-collected samples can be evaluated by providers during sample collection for their suitability and by laboratorians for their biological sufficiency. As we learn more about the capacity of patients to correctly collect specimens and illustrate the use of internal controls to document the biological sufficiency of specimens, there will be opportunities to use SARS-CoV-2 testing in innovative ways to address the COVID-19 pandemic.

## Appendix

Multimedia Appendix 1 Case report forms for self-collection evaluation.

## Abbreviations

COVID-19: coronavirus disease
CRF: case report form
C_t_: cycle threshold
DBS: dried blood spot
EUA: Emergency Use Authorization
FDA: Food and Drug Administration
HIPPA: Health Insurance Portability and Accountability Act of 1996
IgA: immunoglobulin A
IgG: immunoglobulin G
IgM: immunoglobulin M
LDT: laboratory developed test
NPS: nasopharyngeal swab
OPS: oropharyngeal swab
PCR: polymerase chain reaction
PPE: personal protective equipment
qPCR: quantitative polymerase chain reaction
RNase P: ribonuclease P
SARS-CoV-2: severe acute respiratory syndrome coronavirus 2
STI: sexually transmitted infection
