# Supplementary material for: Detection of SARS-CoV-2 RNA and Antibodies in Diverse Samples: Protocol to Validate the Sufficiency of Provider-Observed, Home-Collected Blood, Saliva, and Oropharyngeal Samples
Source: JMIR Public Health Surveill. 2020 Apr 24;6(2):e19054. doi: 10.2196/19054 (PMC7184968; doi:10.2196/19054)
Supplement: Multimedia Appendix 1 [file publichealth_v6i2e19054_app1.pdf]

iCollect CRF 1: Observation Overall

Instructions: Provider should complete this CRF for each participant during their telehealth session. If a participant did not complete their telehealth session, mark “no” for question 1.

Study ID: ☐☐☐☐☐

Clinician last name: ☐☐☐☐☐☐☐☐☐☐☐☐

Combined code: ☐☐☐☐☐☐

First and last initial, last 4 digits of phone number

Date Completed: ☐☐ ☐☐ ☐☐☐☐

Month *Day* *Year*

1. Was observation of specimen collection completed?

☐ Yes ☐ No → a. Why not? ______________________________

____

If no observation done, do not complete remainder of CRF.

2. I observed the participant collect the following specimens:

a. Saliva	☐ Yes	☐ No
b. Oropharyngeal swab	☐ Yes	☐ No
c. Dried blood spot card	☐ Yes	☐ No

3. Indicate whether you are confident the specimens collected below are suitable for laboratory testing.

a. Saliva	☐ Yes	☐ No
b. Oropharyngeal swab	☐ Yes	☐ No
c. Dried blood spot card	☐ Yes	☐ No

4. Date of observation:

☐☐ ☐☐ ☐☐☐☐

Month *Day* *Year*

5. Time observation began:

☐☐ : ☐☐ ☐ AM ☐ PM

hr (0-12) *min (0-59)*

6. Time observation ended:

☐☐ : ☐☐ ☐ AM ☐ PM

hr (0-12) *min (0-59)*

7. Include other relevant notes about the session: ______________________________

____

____

____

iCollect CRF 2: Saliva Specimen

Instructions: Provider should complete this CRF for each participant during their telehealth session. If a participant did not complete the saliva specimen during the session, mark "no" for question 1.

Study ID: ☐☐☐☐☐ Clinician last name: ☐☐☐☐☐☐☐☐☐☐☐☐

Combined code: ☐☐☐☐☐☐ Date Completed: ☐☐ ☐☐ ☐☐☐☐

First and last initial, last 4 digits of phone number *Month* *Day* *Year*

1. Was observation of the **saliva specimen** completed?

☐ Yes ☐ No → a. Why not? ________________________________

____

If no saliva specimen collection, do not complete remainder of CRF.

While observing the collection of the **saliva specimen**, I observed the following:

2. Participant did not eat, drink or smoke immediately before or during the specimen collection. ☐ Yes ☐ No
3. Participant washed hands (or reported washing hands if off camera) before collection. ☐ Yes ☐ No
4. Participant rinsed their mouth with water (or reported rinsing mouth if off camera) before collection. ☐ Yes ☐ No
5. Participant placed lips over funnel and collected saliva sample. ☐ Yes ☐ No
6. Participant filled the tube to the red indicator line. ☐ Yes ☐ No
7. Participant unscrewed the funnel and put on the cap. ☐ Yes ☐ No
8. Participant inverted the vial 20 times. ☐ Yes ☐ No
9. Participant removed the barcode label and applied to the tube. ☐ Yes ☐ No
10. Participant wrote their date of birth on the barcode label. ☐ Yes ☐ No
11. Participant placed the specimen in the biohazard bag and sealed the bag. ☐ Yes ☐ No

12. Notes on saliva specimen collection: ________________________________

____

____

iCollect CRF 3: Oropharyngeal Swab

Instructions: Provider should complete this CRF for each participant during their telehealth session. If a participant did not complete the oropharyngeal swab during the session, mark "no" for question 1.

Study ID: ☐☐☐☐☐ Clinician last name: ☐☐☐☐☐☐☐☐☐☐☐☐

Combined code: ☐☐☐☐☐☐ Date Completed: ☐☐ ☐☐ ☐☐☐☐

First and last initial, last 4 digits of phone number *Month* *Day* *Year*

1. Was observation of the **oropharyngeal swab** completed?

☐ Yes ☐ No → a. Why not? --

If no oropharyngeal swab collection, do not complete remainder of CRF.

While observing the collection of the **oropharyngeal swab**, I observed the following:

2. Participant did not eat, drink or smoke immediately before or during the specimen collection. ☐ Yes ☐ No
3. Participant washed hands (or reported washing hands if off camera) before collection. ☐ Yes ☐ No
4. Participant did not let the swab touch anything before or after sample collection. ☐ Yes ☐ No
5. Participant inserted the swab in their mouth and swabbed each side approximately 20 seconds. ☐ Yes ☐ No
6. Participant placed the swab in the collection tube. ☐ Yes ☐ No
7. Participant broke the swab at the score line. ☐ Yes ☐ No
8. Participant placed the lid on the collection tube and tightened. ☐ Yes ☐ No
9. Participant wrote their date of birth on the tube. ☐ Yes ☐ No
10. Participant placed the specimen in the biohazard bag and sealed the bag. ☐ Yes ☐ No

11. Notes on oropharyngeal swab collection: --

----

iCollect CRF 4: DBS Collection

Instructions: Provider should complete this CRF for each participant during their telehealth session. If a participant did not complete the dried blood spot during the session, mark "no" for question 1.

Study ID: ☐☐☐☐☐ Clinician last name: ☐☐☐☐☐☐☐☐☐☐☐☐

Combined code: ☐☐☐☐☐☐ Date Completed: ☐☐ ☐☐ ☐☐☐☐

First and last initial, last 4 digits of phone number *Month* *Day* *Year*

1. Was observation of the **dried blood spot specimen** completed?

☐ Yes ☐ No → a. Why not? --

If no dried blood spot specimen collection, do not complete remainder of CRF.

While observing the collection of the **dried blood spot specimen**, I observed the following:

2. Participant labeled the DBS card, including name, date of birth and date of collection. ☐ Yes ☐ No
3. Participant did not touch the blood collection paper. ☐ Yes ☐ No
4. Participant washed hands (or reported washing hands if off camera) before collection. ☐ Yes ☐ No
5. Participant cleaned finger with alcohol pad. ☐ Yes ☐ No
6. Participant used lancet on the side of a finger. ☐ Yes ☐ No
7. Participant did not touch the paper while making blood spots. ☐ Yes ☐ No
8. Participant filled spots completely. *(Notice different answer options)* ☐ All ☐ Some ☐ None
9. Participant set the card aside to dry. ☐ Yes ☐ No

10. Number of DBS spots filled: ☐ 0 ☐ 1 ☐ 2 ☐ 3 ☐ 4 ☐ 5

11. Notes on dried blood spot collection: --
